# The “Trade-Off” of Student Well-Being and Academic Achievement: A Perspective of Multidimensional Student Well-Being

**DOI:** 10.3389/fpsyg.2022.772653

**Published:** 2022-03-16

**Authors:** Xiaojun Ling, Junjun Chen, Daniel H. K. Chow, Wendan Xu, Yingxiu Li

**Affiliations:** ^1^School of Educational Sciences, Nantong University, Nantong, China; ^2^Department of Education Policy and Leadership, The Education University of Hong Kong, Hong Kong, Hong Kong SAR, China; ^3^Department of Health and Physical Education, The Education University of Hong Kong, Hong Kong, Hong Kong SAR, China

**Keywords:** student well-being, high school, quantitative, Chinese education, China

## Abstract

Student well-being and its relationships with academic achievement in China have not been well-investigated. This study aimed at investigating student well-being and the trade-off of the well-being and academic achievement with a sample of 1,353 Chinese high-school students from four cities in China during coronavirus disease 2019 (COVID-19) pandemic period. The six dimensions of well-being (academic, psychological, self, physical, social, and spiritual) were utilised to test the relationships with three subjects including Mathematics, English, and Chinese using a quantitative analysis. In this study, the relationships between six dimensions of well-being and three academic subject achievements were tested in one statistical model. Results showed that spiritual well-being was ranked the highest, followed by psychological, physical, self, and social well-being. Students gave the lowest ranking to academic well-being. The two significant paths identified were between spiritual well-being and two subjects, namely, Chinese and Mathematics. It is interesting to note that the other five dimensions of well-being were significantly associated with any subjects and English was not significantly related to any dimensions of well-being in this study. Our findings suggested that policymakers and other stakeholders should avoid an “all or nothing” mindset on practice when considering well-being as a multidimensional construct.

## Introduction

Even though there may be many different visions of “the future we want,” the well-being of society is a shared destination [Organisation for Economic Co-operation and Development (OECD), 2019a]. Students worldwide describe the future that they want, to articulate their hopes, dreams, and the actions needed to attain well-being. Likewise, from surveying parents around the world what they would like to have for their children, it was found that most of them indicated happiness and health, although some also mentioned academic achievement and success (The Children’s Society, 2015; Organisation for Economic Co-operation and Development (OECD), 2019b). By pursuing this better future, the OECD Learning Compass 2030 highlights the element of “Well-being 2030” for our young generation. Research investigations have demonstrated that student well-being is vital for their learning process and for gains to happen (Clarke, 2020; Runions et al., 2021). However, international studies (Roome and Soan, 2019; Dix et al., 2020) have indicated that performativity cultures worldwide are harmful to student well-being. Moreover, it is reported that coronavirus disease 2019 (COVID-19) pandemic is having far-reaching consequences for how students live, work, and connect with one another as well as their well-being over time (Schwartz et al., 2021). It is also argued that during COVID-19 pandemic, students have suffered from increasingly more than the evidence being presented (Organisation for Economic Co-operation and Development (OECD), 2020). This has posed a need to investigate student well-being, especially in high-stakes examination contexts such as China (Chen, 2010; Chen and Teo, 2020). It is well-known that China has a long history of using examinations and tests to select and reward talent and to regard high academic performance on high-stakes examinations as a legitimate, meritocratic basis for upward social mobility regardless of social background. School success is of great importance, leading to Chinese students suffering from great pressure and anxiety while studying for examinations (Chen and Cowie, 2016; Huang and Zhou, 2019).

Student well-being in China seems to be not well-investigated (Cheng et al., 2021). The value of well-being is also reflected by the Programme for International Student Assessment (PISA), as many participating countries wish to know how their students are achieving academically as well as how they conduct their lives on a daily basis. The international data from the PISA 2018 (Organisation for Economic Co-operation and Development (OECD), 2019c) indicate that the level of life satisfaction (59%) that students from China reported is below the OECD average (67%), although students from China (Beijing, Shanghai, Jiangsu, and Zhejiang) outperformed by a large margin over their peers from other participating education systems in Mathematics and Science. Moreover, the average life satisfaction score (6.64 out of 10) is also lower than the average score (7.04) across the OECD countries. When examining the specific ranking score, around 11% of the students from China reported being very satisfied with their lives. Taken together, these studies showed that students in China outperformed in their subjects, but suffered from a low level of well-being. It should be noted that the four provinces/municipalities in eastern China represented in this study could not represent the whole country. However, the size of each province/municipality could be compared to the typical OECD country. In addition, when combining their populations, they exceed 180 million people. Therefore, to some extent, the results of this study could provide implications. Therefore, the need for investigating student well-being in China is even more pressing.

Increasingly competitive education systems and academic accountabilities observed in other localities are expected to show similar difficulties between student performance and well-being academic in China (Chen et al., 2009; Wang et al., 2015). In this context, society attributes enormous value to the academic achievement and experiences of students, which happen simultaneously to them reporting lower levels of well-being and mental health (Boncquet et al., 2020). It has been strongly advised by Clarke (2020) to all the educators to assume a more balanced opinion considering both the realities. It is vital that education systems avoid promoting academic achievement goals without recognising that students’ attainment of such goals is empirically connected to their well-being. Reimagining schools as places where student well-being and learning can be fostered should be the default position held, as opposed to assuming that one must surpass the other. It should be recognised that schools must make provisions for student academic achievement and their well-being (Alivernini et al., 2020). It was found by Bücker et al. (2018) and Rashid and Seligman (2018) that well-being and achievement are mutually synergistic. However, how they connect with each other seems to be closely understood.

Student well-being emerges very early in life and continues developing throughout their years at school (Rothbart et al., 2011). It is particularly important for adolescent student well-being to be investigated, as this is a key developmental transition stage. Students have been asked questions including how happy and satisfied they believe they are in various parts of their lives, how well-connected they believe they are to other people, as well as the aspirations that they hold for their adulthood as common means to understand their state of well-being and relationship with their achievement (Organisation for Economic Co-operation and Development (OECD), 2019b). This is also related to health and behaviour patterns that may continue to persist into adulthood (Patton et al., 2011; Currie et al., 2012). Except for the investigation from the PISA, student well-being at other ages, such as students in high schools, is not well-understood in China. Without sufficient investments in developing capabilities in the present, students may be less likely to enjoy well-being as school-age students and adults as well. Moreover, the existing studies mainly focussed on a single dimension of student well-being, but the need for a nuanced investigation of the multiple dimensions of student well-being has been proposed (Clarke, 2020). Therefore, the aim of this study was to investigate six dimensions of well-being (academic, psychological, self, physical, social, and spiritual well-being) and how they were related to academic achievement (e.g., Mathematics, English, and Chinese) at high schools in China using a relatively large sample from four cities in China.

## Literature Review

Well-being is a dynamic state. Scientists have tried to depict the complexity of the philosophical and humanistic nature of well-being (Berezina et al., 2020; Dix et al., 2020; Hascher and Waber, 2021). Historically, two distinct, yet complementary paradigms have developed in the research field of well-being. One is called “hedonism,” proposed by a Greek philosopher named Aristippus who advocated maximising pleasure in life and believed that happiness was the sum of all the hedonic moments (Ryan and Deci, 2001). The other paradigm was termed “eudemonism” by Aristotle, who identified happiness with living well and the highest good (Ryan and Deci, 2001). Eudemonia is achieved through virtuous actions and the fulfilment of ones’ potential. Extended from this, Waterman (1993) maintained that the eudemonic conception of well-being was related to activities that enable personal growth and improvement. As the concept of well-being evolved, some scholars criticised these two simplified paradigms for failing to uncover the complexity and philosophical concepts of well-being (Berezina et al., 2020). Scholars have proposed to embrace well-being from a multi-directional perspective. Through the integration of the framework for the analysis of student well-being in the PISA 2015 study and results of comprehensive theories and measurements of well-being from a recent review by Cooke et al. (2016), the six domains of student well-being will be presented. They defined that student’s well-being refers to the capabilities that students need in order to live a happy and fulfilling life, which may consist of various functioning (Organisation for Economic Co-operation and Development (OECD), 2017). These included academic, physical, psychological, self, social, and spiritual well-being (Borgonovi and Pál, 2016; Ohrt et al., 2019; Dix et al., 2020; Schwartz et al., 2021). The definitions and content of these six dimensions of student well-being and their relationships with academic achievement are described in the following sections.

Academic well-being encompasses the affective and cognitive self-concept, in addition to liking school subjects and matters in school. It refers to the foundations, knowledge, and skills that students possess in order to effectively participate as lifelong learners in today’s society and be effective workers and engaged members (Borgonovi and Pál, 2016). Research studies including Elovainio et al. (2011), Tuominen-Soini et al. (2011), and Fiorilli et al. (2017) have consistently shown a relationship existing between student academic well-being and academic achievement. Academic well-being can be a reflection on the academic engagement students have including how much time, effort, and energy they make in their work as well as the contribution that they make, their understanding and what they have gained as a result of their schoolwork. Learning time quickly passes when students have full engagement with their studies. Their sense of self-efficacy may also improve. In Miller et al. (2013) study, for example, the conception of academic buoyancy was used for well-being to be accessed at school. The authors found a strong positive relationship that was apparent with the academic achievement of the students. It was confidently concluded by Rimpelä et al. (2020) that students’ overall development is promoted by academic well-being including academic outcome.

Psychological well-being comprises three aspects: positive emotions, self-worth, and self-esteem regarding performance. Included are the evaluations students have of themselves, opinions about their lives, school engagement as well as future ambitions and goals. According to Ryff and Singer (2006), an individual who possesses a high level of psychological well-being endeavours to have an aim in their life (purpose in life), continuously experiences personal development (personal growth), and has the impression of being able to influence their environment (environmental mastery). When Ryff and Singer (2006) were examining the relationship that occurs between psychological well-being and academic achievement, they demonstrated that psychological well-being is consistently associated with educational attainment. Through a review of relevant publications, it was discovered by Gräbel (2017) that students’ psychological well-being is strongly related to their academic achievement.

Self well-being primarily includes three aspects of self, namely, self-confidence, self-esteem, and self-worth. Self-worth is concerned with an individual recognising their capacities and having confidence in them as well as having the belief that they are of value and benefit to other people; self-esteem includes the two aspects of self-worth and self-comment (Neff, 2011). Students who have healthy self-esteem usually have strong belief in themselves, are unafraid to confront any challenges they may have in life and make attempts, will not be frustrated for long periods even when they experience times of failure and can keep a positive viewpoint on their life in the future. Having a high level of self well-being does not equate to an individual being arrogant or having omnipotence but, instead, having acknowledegment of their values and weaknesses. It has been reported by Yu et al. (2018) that it is more likely students who have a good level of self well-being will take risks and challenges in their lives. For example, students who have high self-esteem and self-worth may participate actively in their classes and answer questions, even when they have doubts that their answers are correct. Also, as reported by Fairlamb (2020), there is a greater likelihood that such students will establish healthy interpersonal relationships, have stronger problem-solving skills and higher creativeness, claimed to be related to achieving higher academic outcomes. Furthermore, in science and health, student achievement and confidence scale scores have been explored in relation to “Belonging at School” scale scores. In their study, Gilmore and Asil (2019) examined student achievement and confidence scale scores in the subjects of Mathematics and Social Studies in relation to the aspect of “Feeling Safe at School.”

Physical well-being is concerned with vitality and physical health, focussing on the health status of students, their engagement in physical activities and exercise and adopting healthy eating habits. Research studies (Basch, 2011; Castelli et al., 2014) have reported positive relationships existing amid academic success, cognition, youth physical fitness, and physical well-being. Specifically, several researchers (Castelli et al., 2007; Van Dusen et al., 2011; Srikanth et al., 2015) have shown that students who possess higher physical fitness levels are more likely to be successful in their academic work. Castelli et al. (2014) have shown that a direct, positive link exists between physical fitness and executive function and this can, in the long run, have an impact on achievement. Furthermore, although the literature associated with physical fitness is not very strong, it has been proven nevertheless by scientists (Centeio et al., 2018; McPherson et al., 2018) that a positive relationship exists between students undertaking regular moderate to vigorous physical fitness and their school academic performance. Student levels of physical fitness in relation to their academic test scores were examined by Donnelly and Lambourne (2011). They reported that the children who undertook regular moderate physical fitness through classroom interventions scored significantly higher on their school achievement tests. Also, a direct relationship was found in a recent research study (Centeio et al., 2020) in which physical well-being was assessed by physical activity enjoyment, healthy self-concept, and academic achievement.

Social well-being has been acknowledged by the WHO (World Health Organization [WHO], 1948) as being a central element of a person’s overall well-being. Social well-being has been denoted by Fattore and Mason (2017) and Ohrt et al. (2019) as the ability for an individual to develop positive social relationships with others when playing various social roles and perceiving their social life at school; for example, the establishment of good relationships with members of their family and friends, receiving support from them and, as a result, achieving happiness in their lives. Social well-being encompasses social self, namely, the social roles of an individual in varying situations. This may be indicated by adopting a positive outlook toward others, belief in the growth of society, having an understanding of others in society, participation within different aspects of society and identifying with society. Social well-being is significant as a whole to students, school, community as well as society. Students play many roles in their lives. At home, they are children; in school, they are students; and when communicating with peers, they are assuming the role of friends. With a high level of social well-being, they have the skills and knowledge of how to interact with others and after receiving feedback on their actions, adjust themselves as required. It has been reported in research studies (Cicognani, 2014; Gräbel, 2017; Samad et al., 2019) that during the process of playing various roles and their interactions with others, students may also make improvements in their academic achievement.

Spiritual well-being connects spirituality and health. It has been conceptualised by Moberg (1971) that spiritual well-being has a personal connection with God, life meaning and satisfaction without there being any religious meanings. The spiritual well-being model proposed by Fisher (1998) and subsequent updated version contains the fields of personal well-being (how an individual intra-relates with oneself), communal (the quality of inter-personal relationships), environmental (the care and nurture one have for the physical and biological world), and finally transcendental (the relationship a person has with something or someone outside the human level). According to Fisher (2013), this model indicates the importance attached to the development of joy in life, love of/for others and having peace with God as being the perfect scenario for spiritual well-being to be built as well as the enrichment of life experience. Having connections with spiritual wellness plays a central position in the maintenance and promotion of an individual’s mental health and spiritual well-being, thus indicating an increasing a mental health and improving the experiences that students encounter throughout their lives (Lu et al., 2019). When considering the notion of achievement, research (Ko, 2016) has reported a positively significant relationship existing between student spiritual well-being and their performance academically.

To sum up, although, with inconsistent results, most studies found a positive “trade-off” existing between well-being and achievement (Clarke, 2020). However, the majority of these studies only measured a single dimension of student well-being and its relationship with academic outcomes within a single subject. This study aimed at investigating the multiple dimensions of student well-being with three different school subjects. Two research questions were proposed as follows:

1. What is the situation of the six dimensions of student well-being, namely, academic well-being, psychological well-being, self-well-being, physical well-being, social well-being and spiritual well-being?
2. What are the relationships of the six dimensions of student well-being with their achievement of Mathematics, English, and Chinese?

The hypothesis was generated based on the second research question: Six dimensions of student well-being significantly related to their academic achievement of Mathematics, English, and Chinese.

## Materials and Methods

### Sample

This study was based on a convenience sample from 12 high schools in four cities in a northern province in China. The education resources and educational performance of this province are relatively on the average for the whole of China, meaning they are considerably lower than that seen in the wealthy coastal cities of eastern and southern China. Although generalisability to the whole population of Chinese students is doubtful given the sampling from one province, the sample may be indicative of student well-being, at least in the targeted province of China. The questionnaire was distributed to 1,353 Chinese students in their first year and second year in December 2020 during COVID-19 pandemic period.

A total of 1,014 valid questionnaires were returned giving a response rate of 81.6%. Among the students, 585 questionnaires were in their first year and 429 questionnaires were in their second year. The gender distribution was almost even with 541 girls (53.4%) and 470 boys (46.4%) and their average age range between 16 and 17 years old. In terms of the educational background of the parents, more than half of them were below Bachelor’s degree (68.5% for mothers and 66.2% for fathers), followed by Master’s degree (26% for mothers and 27.7% for fathers).

The research ethics approval has been obtained from the university of the second author. After briefing each principal on the project, researchers obtained permission to recruit volunteer student participants within each school. Once the principal agreed to participate, teacher volunteers were recruited. Teachers were asked to distribute student participant information sheets and the questionnaire to the students in their classrooms. Students were asked to fill in the questionnaire in a quiet place and return completed questionnaires within 4 weeks directly to the research team using pre-addressed, stamped envelopes, or to a drop-box at school within 2 weeks (Table 1).

**TABLE 1:** Demographic information of the participants.

Demographic	Total	Percent (%)
**Sex**		
Female	541	53.5%
Male	470	46.4%
**Grade**		
First year	585	57.7%
Second year	429	42.3%
**Age**		
≤14	7	0.7%
15	115	11.5%
16	532	53%
17	323	32.2%
≥18	27	2.7%
**Father’ educational background**		
Below bachelor degree	576	66.2%
Bachelor degree	241	27.7%
Master degree	23	2.6%
Doctor degree or above	31	3.6%
**Mother’ educational background**		
Below bachelor degree	592	68.5%
Bachelor degree	225	26%
Master degree	16	1.8%
Doctor degree or above	32	3.7%

### Measures

In this study, six scales of different dimensions in well-being were selected to measure student well-being, namely, academic well-being, psychological well-being, self-well-being, physical well-being, social well-being, and spiritual well-being. A 6-point frequency rating scale was used in all the scales.

First, academic well-being was measured by four items selected from the Self-Description Questionnaire II (Marsh, 1990), which consists of subject and school liking. Second, psychological well-being consisted of four items drawn from the positive emotion scale out of the Positive Emotion, Engagement, Relationships, Meaning, Achievement (PERMA) (Seligman, 2011) and the Life Resilience Scale (Martin and Marsh, 2008). Psychological well-being was comprised of positive emotions, optimism, and resilience. Third, four items were selected from the Self-Description Questionnaire III Adjusted (Marsh, 1992) to assess student self-well-being with two dimensions, namely, self-worth and self-esteem. Fourth, physical well-being consisted of four items from the Vitality Scale (Ryan and Frederick, 1997) measuring vitality, physical health, affective of physical activity, and cognitive of physical activity. Fifth, the four items of social well-being were taken from the Relations Scale by Ryan (1995) measuring family relations, community relations, school relations, and peer relations. Sixth, four items were selected from the Spiritual Health and Life-Orientation Measure 2 (SHALOM 2) by Fisher (2013) covering the personal, communal, environmental, and transcendental dimensions.

#### Students’ Academic Achievement

Student academic achievements were collected through the scores of the Chinese, Mathematics, and English subjects. The full mark of the three subjects was classified into five rankings, i.e., *outstanding (135–150)*, *very good (120–134)*, *good (105–119)*, *pass (90–104), and fail (0–89)*. In terms of the Chinese subject, the findings demonstrated that the students gained an equal percentage (26, 2.8%) for outstanding and very good, 12.1% (111) for good, more than half (485, 52.9%) for pass, and less than one-third (268, 29.3%) for fail. For Mathematics, 35 (3.8%) of the students gained outstanding, 37 (4%) gained very good, 73 (8%) gained good, 288 (31.5%) gained pass, and over half (482, 52.7%) gained fail. The result of the English subject showed 35 (3.8%) gained outstanding, 43 (4.7%) gained very good, 131 (14.3%) gained good, 324 (35.5%) gained pass, and 380 (41.6%) gained fail.

#### Analysis Procedure

A cross-validation method (Gerbing and Hamilton, 1996) with exploratory factor analysis (EFA) and confirmatory factor analysis (CFA) was utilised to generate and, then, to confirm the model. More specifically, EFA was used on one randomly selected half of the sample (507) to generate new exploratory models of six dimensions of well-being and CFA was used on the other half (507) to test the replicability of the modified models of the six dimensions of well-being. An advantage of the cross-validation method is that it allows the testing and modification of the exploratory model on an independent subset of the sample (Gerbing and Hamilton, 1996).

There were five steps involved in the data analysis procedure. First, missing data were calculated with the expectation maximisation algorithm performed in SPSS software version 26. Second, EFA with maximum likelihood estimation and oblique rotation was employed to test the six models of well-being performed in SPSS software version 26. Items were removed that had loadings lower than 0.30 on their intended conceptual factors or which did not match logically with other items in the same factors during EFA. During this process, 24 out of 38 items divided into six dimensions were kept for further analysis. Third, reliability analysis was used to calculate the Cronbach’s coefficient of each variable. The descriptive statistics including mean (M) and SD were also obtained. Fourth, six dimensions of well-being with four items were individually tested with CFA performed in Mplus and all the models with a good model fit were subsequently tested in a CFA model. The reason for doing this was to keep a tidy model for each of the six dimensions. In other words, we tended to keep four items for each dimension. Fifth, a multidimensional model of well-being with six dimensions was tested using structural equation modelling (SEM).

The following criteria were employed during the analysis: (1) first, factors had to have three or more conceptually aligned items; (2) items with regression loadings of >0.30 and (3) all the cross-loadings had to be <0.30 (Bandalos and Finney, 2010). In line with current practice (Marsh et al., 2004), a multi-criteria acceptable model fit was: (1) statistically non-significant χ^2^/df (*p* > 0.01), (2) gamma hat and comparative fit index (CFI) ≥ 90; and (3) both the root mean square error of approximation (RMSEA) and standardized root mean square residual (SRMR) ≤ 0.08. Models that met these criteria were not rejected because the specified model was not significantly different from the real patterns of covariance and variance in the data.

## Results

It was significant to measure the psychometric characteristics of each scale separately (i.e., the measurement model). If each component of the model had poor properties, this would have affected the quality of the conjoint model. For each instrument, descriptions were given for the model fit characteristics, the items belonging to each factor and the strength of their loading on the factor (Table 2). Descriptive statistics including means (*M*) and *SDs* reliability are shown in Table 3. Besides, the inter-correlations among the factors (Table 4) and the correlation between the six dimensions of well-being and student achievement are discussed.

**TABLE 2:** Multidimensional well-being items and factor loadings.

Scale and items	M (SD)	Factor loading
Multidimensional well-being model	4.50 (1.01)	
Academic well-being	4.15 (1.29)	
1. I like most school subjects.	4.45 (1.39)	0.72
2. I enjoy most school subjects.	4.15 (1.50)	0.87
3. I look forward to going to school.	4.07 (1.64)	0.77
4. Things in most school subjects are easy for me.	3.95 (1.58)	0.85
Psychological well-being	4.56 (1.22)	
5. I trust my future will turn out well.	4.75 (1.34)	0.81
6. I expect good things to happen to me.	4.57 (1.43)	0.87
7. I enjoy life.	4.52 (1.37)	0.85
8. I have a lot of fun.	4.41 (1.48)	0.82
Self well-being	4.50 (1.28)	
9. I like myself.	4.54 (1.39)	0.90
10. I feel good about myself.	4.49 (1.40)	0.85
11. If I try hard I can do almost anything I want to do.	4.48 (1.44)	0.90
12. I do things as well as most people.	4.47 (1.45)	0.83
Physical well-being	4.51 (1.11)	
13. I am good at most sports and games.	4.68 (1.29)	0.68
14. My body is healthy.	4.60 (1.41)	0.70
15. I do not easily get tired out.	4.41 (1.40)	0.74
16. I have lots of energy.	4.34 (1.45)	0.78
Social well-being	4.45 (1.29)	
17. I feel my friends at school care about me.	4.47 (1.37)	0.71
18. I feel close and connected with my friends at school.	4.45 (1.42)	0.89
19. I like being with my friends at school.	4.45 (1.36)	0.86
20. When I am with my friends at school, I feel like I belong.	4.44 (1.43)	0.84
Spiritual well-being	4.88 (1.07)	
21. I develop trust between individuals.	4.89 (1.20)	0.84
22. I develop respect for others.	4.89 (1.20)	0.91
23. I develop awe at a breath-taking view.	4.88 (1.25)	0.45
24. I develop kindness toward other people.	4.86 (1.24)	0.85

**TABLE 3:** Means, SDs, and Cronbach α of six dimension of well-being.

Multidimensional well-being model	M	SD	Cronbach α
	
	4.50	1.01	0.96
Spiritual well-being	4.88	1.07	0.90
Psychological well-being	4.56	1.22	0.89
Physical well-being	4.51	1.11	0.81
Self well-being	4.50	1.28	0.92
Social well-being	4.45	1.29	0.95
Academic well-being	4.15	1.29	0.87

**TABLE 4:** Student’s well-being intercorrelations and correlations.

Well-being scale	Academic	Psychological	Self	Physical	Social	Spiritual
Academic	-					
Psychological	0.66**	-				
Self	0.66**	0.76**	-			
Physical	0.63**	0.74**	0.80**	-		
Social	0.56**	0.69**	0.60*	0.64**	-	
Spiritual	0.45**	0.51**	0.48**	0.57**	0.52**	-

**Correlation is significant at the 0.01 level (2-tailed).

### Measurement Models of the Six Dimensions

#### Academic Well-Being

The 4-item academic well-being model was further determined by CFA and a sufficient model fit was identified (χ^2^ = 4.87; df = 2; χ^2^/df = 2.44; CFI = 0.997; Tucker-Lewis index (TLI) = 0.991; RMSEA = 0.057 and SRMR = 0.012). The factor loadings of all the items were greater than 0.71 (Table 2). The four items mainly focussed on student interest and statement of being in school, their liking of the subjects being taught and their efficacy in learning the subjects. The descriptive statistics showed that the mean value of academic well-being was 4.15, which was ranked lower than the other five scales. Besides, the value of SD was 1.29. It is noted that the reliability of academic well-being revealed a satisfactory level of internal consistency of 0.87.

#### Psychological Well-Being

The 4-item psychological well-being model revealed an adequate model fit in CFA, i.e., χ^2^ = 5.08; df = 2; χ^2^/df = 2.54; CFI = 0.997; TLI = 0.992; RMSEA = 0.059 and SRMR = 0.009. All the factor loadings of the items were above 0.79 (Table 2). The items identified the positive emotion and resilience of students like “I trust my future will turn on well” and “I expect good things to happen to me.” According to descriptive statistics, psychological well-being was the second most frequent well-being in all the scales (*M* = 4.56, *SD* = 1.22) and it also demonstrated the high factor reliability of 0.89.

#### Self-Well-Being

The four items constituted the new self-well-being and exhibited a good model fit in CFA (χ^2^ = 7.88; df = 2; χ^2^/df = 3.94; CFI = 0.996; TLI = 0.987; RMSEA = 0.08 and SRMR = 0.009). Each item demonstrated a high factor loading with greater than 0.82 (Table 2). This scale regards student cognition of their self-worth and self-esteem (i.e., “I like myself” and “If I try hard, I can do almost anything I want to do”). In terms of descriptive statistics, self-well-being ranked 4th frequent in six well-being scales (*M* = 4.50, *SD* = 1.28). The Cronbach’s α for the inter-connectivity of self-well-being showed high reliability of 0.92.

#### Physical Well-Being

Similar to the above scale, the confirmed measurement model of physical well-being included four items and CFA showed an acceptable model fit (χ^2^ = 6.44; df = 2; χ^2^/df = 3.22; CFI = 0.992; TLI = 0.977; RMSEA = 0.071 and SRMR = 0.015). All the factor loadings of the items were higher than 0.70 (Table 2). The physical well-being focussed on student evaluation on health issues and the participation of sports games such as “I have lots of energy” or “I am good at most sports and games.” The descriptive statistics showed the mean value of physical well-being was ranked as 3rd (*M* = 4.51, *SD* = 1.11) in all the well-being scales. The reliability indicated a high internal consistency of 0.81.

#### Social Well-Being

A good model fit was evaluated when CFA was performed on 4 items of the social well-being, i.e., χ^2^ = 21.98; df = 2; χ^2^/df = 10.993; CFI = 0.982; TLI = 0.946; RMSEA = 0.0.15 and SRMR = 0.021. Although RMSEA was not good, all the other indicators were good enough. Hence, we kept this current model for further analysis at this stage. All the factor loadings of the items were above 0.71 (Table 2). The items of this scale referred to student identity toward relationships with their classmates. Descriptive statistics revealed that the mean value was 4.45 and the value of the SD was 1.29. This scale presented good reliability on the whole with 0.95.

#### Spiritual Well-Being

The 4-item spiritual well-being model was further determined by CFA and the result of model fit was showed χ^2^ = 3.14; df = 2; χ^2^/df = 1.57; CFI = 0.999; TLI = 0.996; RMSEA = 0.036 and SRMR = 0.01. Each item demonstrated a high factor loading greater than 0.45 (Table 2). The four items mainly indicated students’ personal awareness and the sense of identity with other person and the nature. According to the descriptive statistics, the frequency of spiritual well-being ranked the top (*M* = 4.88, *SD* = 1.07). Meanwhile, the reliability of this scale was the highest within all the six well-being scales with 0.90.

### Multidimensional Well-Being Model

The revised multidimensional well-being measurement model regarded six kinds of well-being scales mentioned above as six inner-correlated factors and included 24 items in total. CFA revealed an acceptable model fit (χ^2^ = 888.86; df = 237; χ^2^/df = 3.75; CFI = 0.922; TLI = 0.909; RMSEA = 0.079 and SRMR = 0.043). The factor loadings of the items in all the six factors ranged from 0.45 to 0.91.

According to the descriptive statistics, it was revealed that spiritual well-being ranked as the most frequent well-being (*M* = 4.88 for both, *SD* = 1.07) (Table 3). In contrast, academic well-being was the least frequent (*M* = 4.15, *SD* = 1.29). All of these illuminated students focussed on both the mind and body well-being in their life, but were shown to be relatively weak in academic well-being. It is noted that the correlations between the factors of multidimensional well-being measurement ranged from small (*r* = 0.45) to large (*r* = 0.80) in Table 4 with an average value of 0.62. Self well-being and physical well-being showed the highest correlation (*r* = 0.80^**^, *p* < 0.01), while all the factors in multidimensional well-being measurement demonstrated a positive correlation with each other. The alpha value of the six factors ranged from 0.81 to 0.95 and the reliability of the whole multidimensional well-being was 0.96, which reflected a high level of internal consistency.

### Relationship Between Well-Being and Student Achievement

**FIGURE 1:**
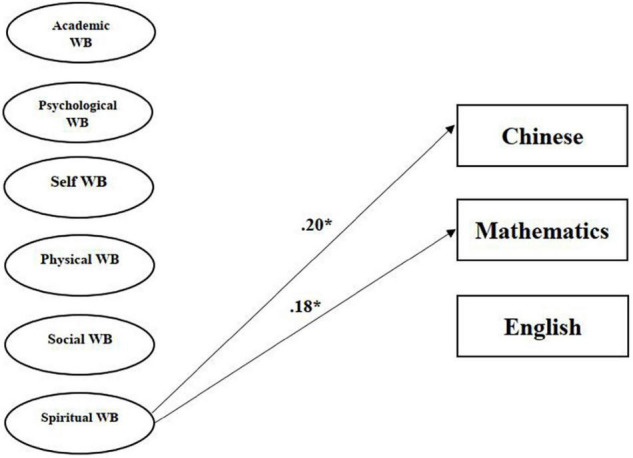
The model of Multiple Well-being (WB) and student achievement. Intercorrelations and error terms were removed for simplicity. *Regression path is significant at the 0.05 level (2-tailed).

The relationship between student well-being and achievement was tested using SEM in Mplus. Student’s achievement was divided into three subjects: Chinese, Mathematics, and English. The final model in which all the paths from multidimensional well-being to student achievement was tested using standardised estimates based on the assumptions. After removing statistically and non-significant paths, the revised model indices showed a great model fit, i.e., χ^2^ = 966.21; df = 291; χ^2^/df = 3.32; CFI = 0.935; TLI = 0.922; RMSEA = 0.072 and SRMR = 0.04 (Figure 1). Generally, this model demonstrated that spiritual well-being was only positively associated with student achievement, while there were no significant relationships between student achievement and the other five kinds of well-being (academic, psychological, physical, self, and social well-being). There are a total of two statically significant paths out of a possible 18 from six multidimensional well-being factors to three achievement factors. The two beta values of all the paths were 0.18 and 0.20, i.e., spiritual well-being was more associated with Chinese and Mathematics, respectively. To conclude, student achievement, especially in Chinese and English grades, it can be generally predicted by their spiritual well-being.

## Discussion

This study responded to two research questions, namely, the situation of student well-being and the relationship between student well-being and their academic achievement (e.g., Mathematics, English, and Chinese) using a large sample size of students from China. The hypothesis on the significant relationships between the six dimensions of well-being and academic subject was also tested. Generally, spiritual well-being ranked the highest one followed by psychological well-being, physical well-being, self-well-being, social well-being, and academic well-being. As for the trade-off between student well-being and achievement, a total of two statically significant relationships from six multidimensional well-being factors to three achievement factors, i.e., spiritual well-being affecting Chinese and Mathematics, respectively. This section outlines the major findings discussed with other relevant literature.

Spiritual well-being ranked the highest, followed by psychological, physical, self, and social well-being. Students gave the lowest ranking to academic well-being. All of these illuminated the fact that the students were focussed on both the mind and body well-being in their lives, but showed being relatively weak in their academic well-being. This low level of academic well-being may be caused by the high-stakes examination atmosphere that exists in Chinese high schools (Chen and Brown, 2016, 2018) and high expectations on student academic achievement from parents and schools (Chen and Day, 2014; Chen, 2016). Furthermore, in this study, social well-being was ranked the second lowest well-being. Social well-being was reported in a study conducted by Samad et al. (2019) as surrounding social self, which, in differing circumstances, can also be considered to be an individual person’s social roles. Possessing a high degree of social well-being, students will have the necessary knowledge and skills to be able to interact with others more positively that could, possibly, strengthen their academic achievement (Gräbel, 2017; Cheng et al., 2021). A research study (Lu, 2010) conducted in Hong Kong also accentuated the paramount priority of relationships on Chinese well-being due to the importance of inter-dependence that is in apparent within Chinese society. Past research conducted on students living in Western countries has reported the importance and effect of peer relationships on student well-being, whereas in contrast within the Chinese context, social failure has been found to be more catastrophic. China, being a collectivist nation, could possibly be a reason for this notion. “I” and “true self” continue to be hazy concepts for Chinese students for them to be able to show appreciation and that they are innately bound to others. As a consequence, the Chinese model of well-being is considered to be socially oriented and, thus, more concerned about the community welfare and role responsibilities of individual people (Lu, 2010). In these types of situations, students will aim to achieve the very best of their ability and adjust if and where they need to in order to be accepted into school life, family, and society thus, striving for harmonious relations with the environment and, simultaneously, looking after their well-being. However, when negative relations exist at school, for example, bullying, this interpersonal harmony will be threatened thus, indicating a collapse of role-playing within the school context. As a result, student well-being will be reduced, but also dissatisfaction will be aroused for school, occasionally causing them to experience psychological problems (Cheng et al., 2021). This may also give reason for the non-significant relationship existing between a student’s social well-being and their school academic attainment.

It is noted that the correlations between the factors of multi-dimensional well-being measurement ranged from small (*r* = 0.45) to large (*r* = 0.80) with an average correlation value of 0.62, indicating these six had something in common but were still varied. Self-well-being and physical well-being showed the highest correlation, while all of the factors in the multi-dimensional well-being measurement demonstrated a positive correlation with each other. The alpha value of the six factors had high reliabilities, reflecting a high level of internal consistency. These findings not only provide relatively rich information on the current situation, but also the sequence of the six dimensions of student well-being. In addition, the inter-correlations between the different types of well-being demonstrated that these types of well-being could affect each other, which offers an alternative way for enhancing student well-being as a whole rather than separately.

The hypothesis on the significant relationships between the six dimensions of well-being and academic subjects was also tested. It was found that spiritual well-being was positively and significantly associated with Chinese and Mathematics achievement, but unfortunately, the other five dimensions of well-being did not have significant relationships with any subject achievement. These evidences yield interesting comparisons with the existing literature. First, the relationship between academic well-being and Mathematics and Chinese identified in this study is not consistent with those from previous studies (Miller et al., 2013; Rimpelä et al., 2020). Second, scholars (Ryff and Singer, 2008; Gräbel, 2017) also found the association between psychological well-being and student achievement. However, the students in this study did not neither report this connection. This may echo Heller-Sahlgren’s (2018) comment on the complexity of the relationship between psychological well-being and achievement that student psychological well-being and achievement do not go arm-in-arm. Third, Gilmore and Asil (2019) found a positive connection between students’ academic scores in Mathematics and Social Studies and their self-confidence. However, this study did not acknowledge this relationship with any subject out of three. Fourth, Centeio et al. (2018) reported that students’ physical fitness directly impacted their success in, but not in reading. This is also not the case in this study. Fifth, likewise, social well-being was not associated with any subject, which is distinct from those from other relevant literature (Ko, 2016; Samad et al., 2019). Finally, as mentioned above, the only two significant paths identified in this study are between spiritual well-being and Chinese and Mathematics, which echo results in the literature (Cicognani, 2014; Gräbel, 2017). These findings are distinct from those from relevant literature (Cicognani, 2014; Ko, 2016; Gräbel, 2017; Samad et al., 2019). These mixed evidences concerning the relationships between different dimensions of student well-being and academic achievement offer insights in promoting well-being simultaneously and aiming for achievement improvement. However, the reasons behind these mixed evidences in this study need further investigations (Clarke, 2020).

## Implications

This study has some important implications. First, it will contribute to the quantitative understanding of the relationships between student well-being and academic achievement. In this sense, this study promotes the knowledge construction of student well-being under the relational framework with academic achievement (e.g., spiritual well-being and Chinese and Mathematics). Second, this study highlights the complexity of student well-being from the multi-dimensional perspective embedded in China context. This result provides implications to stakeholders to pay special attention to the lower levels of well-being (e.g., academic well-being and social well-being) and the higher levels of well-being (e.g., spiritual well-being and psychological well-being) in the China context and other contexts as well, especially as the data were collected during COVID-19 pandemic.

## Future Studies

It is a standing dish for the argumentation of these mixed evidences regarding the “trade-offs” between student well-being and academic achievement (Clarke, 2020). For example, it was proposed by Heller-Sahlgren (2018) of an expected connection existing between student well-being and their achievement in academic subjects. Employing the PISA 2012 data, it has been argued by Heller-Sahlgren that student psychological well-being and achievement do not go arm-in-arm. This suggests a need for policymakers to decide which of these are maintained as considered to be the priority. Grounded on the arguments put forward by Clarke (2020) commented “far from being incompatible, student well-being and achievement are positively associated” (p. 263). It is imperative that the well-being achievement relationship is further explored in future research for more in-depth scrutiny. Careful disentangling of the many elements of well-being and other thoughts is required including the objective operationalisation of the achievement and investigation of differences that exist developmentally. Under COVID-19 pandemic, this is especially apparent.

It is of importance to review the evidence and “unknowns” that are in existence within the well-being achievement relationship. When filling these gaps, there is a need for researchers to consider influences at different levels, namely, national, home, and school with regard to the trade-offs in existence between achievement and student well-being. It is impossible to fully understand student’s well-being relative to their achievement and not simultaneously to have a wider mindfulness regarding the corresponding school, home, and societal networks in which they are positioned (Clarke, 2020). There is a need for increased consideration and understanding of the individual, home, and school settings in which students are rooted and how the individual needs of students are being addressed. It is clearly a requirement for there to be sophisticated research designs and methodologies in order to accommodate for the difficulties of the well-being-achievement relationship that exist. It would be prudent for researchers to contemplate all of these factors before embedding the notion that policymakers should abandon children’s well-being in favour of achievement usually being prioritised.

## Limitations

Despite these contributions, the following limitations are addressed. The first limitation is that the structural relationships identified in this study may be sensitive in different cases, as this study is cross-sectional. The second limitation is that this study did not investigate influential factors (e.g., individual, home, and school). Third, the results from this study may be any bias caused by self-reporting data.

To conclude, driven by the call for the investigation on the multi-dimensions of student well-being, this study coins the nuance of “trade-off” of well-being-achievement in one model. Bearing in mind a wider current of thinking in education whereby pitting achievement goals against the goals of well-being, the relationship between student well-being and achievement is compatible, but not straightforward. Therefore, when making policy recommendations, researchers should avoid “all or nothing” thinking, which lures governments into false dichotomies (Clarke, 2020, p. 623).

## Data Availability Statement

The raw data supporting the conclusions of this article will be made available by the authors, without undue reservation.

## Ethics Statement

The studies involving human participants were reviewed and approved by Education University of Hong Kong. Written informed consent for participation was not required for this study in accordance with the national legislation and the institutional requirements.

## Author Contributions

XL set up the framework, revised the manuscript, and collected data. JC wrote the first draft of the manuscript. DC revised the manuscript and fund support. WX search literature and wrote the draft of literature. YL did data analysis under support of the JC. All authors contributed to the article and approved the submitted version.

## Conflict of Interest

The authors declare that the research was conducted in the absence of any commercial or financial relationships that could be construed as a potential conflict of interest.

## Publisher’s Note

All claims expressed in this article are solely those of the authors and do not necessarily represent those of their affiliated organizations, or those of the publisher, the editors and the reviewers. Any product that may be evaluated in this article, or claim that may be made by its manufacturer, is not guaranteed or endorsed by the publisher.
